# Diagnostic value of artificial intelligence-based software for the detection of pediatric upper extremity fractures

**DOI:** 10.1007/s00330-025-11947-w

**Published:** 2025-08-23

**Authors:** Federico Mollica, Corona Metz, Matthias Stephan Anders, Kim Kathrin Wismayer, Andrea Schmid, Stefan M. Niehues, Simon Veldhoen

**Affiliations:** 1 Department of Radiology, Pediatric Radiology, Charité – Universitätsmedizin Berlin, corporate member of Freie Universität Berlin and Humboldt-Universität zu Berlin,; 2 Department of Radiology, Caritas Gesundheit Berlin, Berlin, Germany

**Keywords:** Machine learning, Deep learning, Radiograph, Fracture, Pediatric

## Abstract

**Objectives:**

Fractures in children are common in emergency care, and accurate diagnosis is crucial to avoid complications affecting skeletal development. Limited access to pediatric radiology specialists emphasizes the potential of artificial intelligence (AI)-based diagnostic tools. This study evaluates the performance of the AI software BoneView® for detecting fractures of the upper extremity in children aged 2–18 years.

**Materials and methods:**

A retrospective analysis was conducted using radiographic data from 826 pediatric patients presenting to the university’s pediatric emergency department. Independent assessments by two experienced pediatric radiologists served as reference standard. The diagnostic accuracy of the AI tool compared to the reference standard was evaluated and performance parameters, e.g., sensitivity, specificity, positive and negative predictive values were calculated.

**Results:**

The AI tool achieved an overall sensitivity of 89% and specificity of 91% for detecting fractures of the upper extremities. Significantly poorer performance compared to the reference standard was observed for the shoulder, elbow, hand, and fingers, while no significant difference was found for the wrist, clavicle, upper arm, and forearm. The software performed best for wrist fractures (sensitivity: 96%; specificity: 94%) and worst for elbow fractures (sensitivity: 87%; specificity: 65%).

**Conclusion:**

The software assessed provides diagnostic support in pediatric emergency radiology. While its overall performance is robust, limitations in specific anatomical regions underscore the need for further training of the underlying algorithms. The results suggest that AI can complement clinical expertise but should not replace radiological assessment.

**Key Points:**

***Question*** *There is no comprehensive analysis of an AI-based tool for the diagnosis of pediatric fractures focusing on the upper extremities.*

***Findings*** *The AI-based software demonstrated solid overall diagnostic accuracy in the detection of upper limb fractures in children, with performance differing by anatomical region.*

***Clinical relevance*** *AI-based fracture detection can support pediatric emergency radiology, especially where expert interpretation is limited. However, further algorithm training is needed for certain anatomical regions and for detecting associated findings such as joint effusions to maximize clinical benefit.*

**Graphical Abstract:**

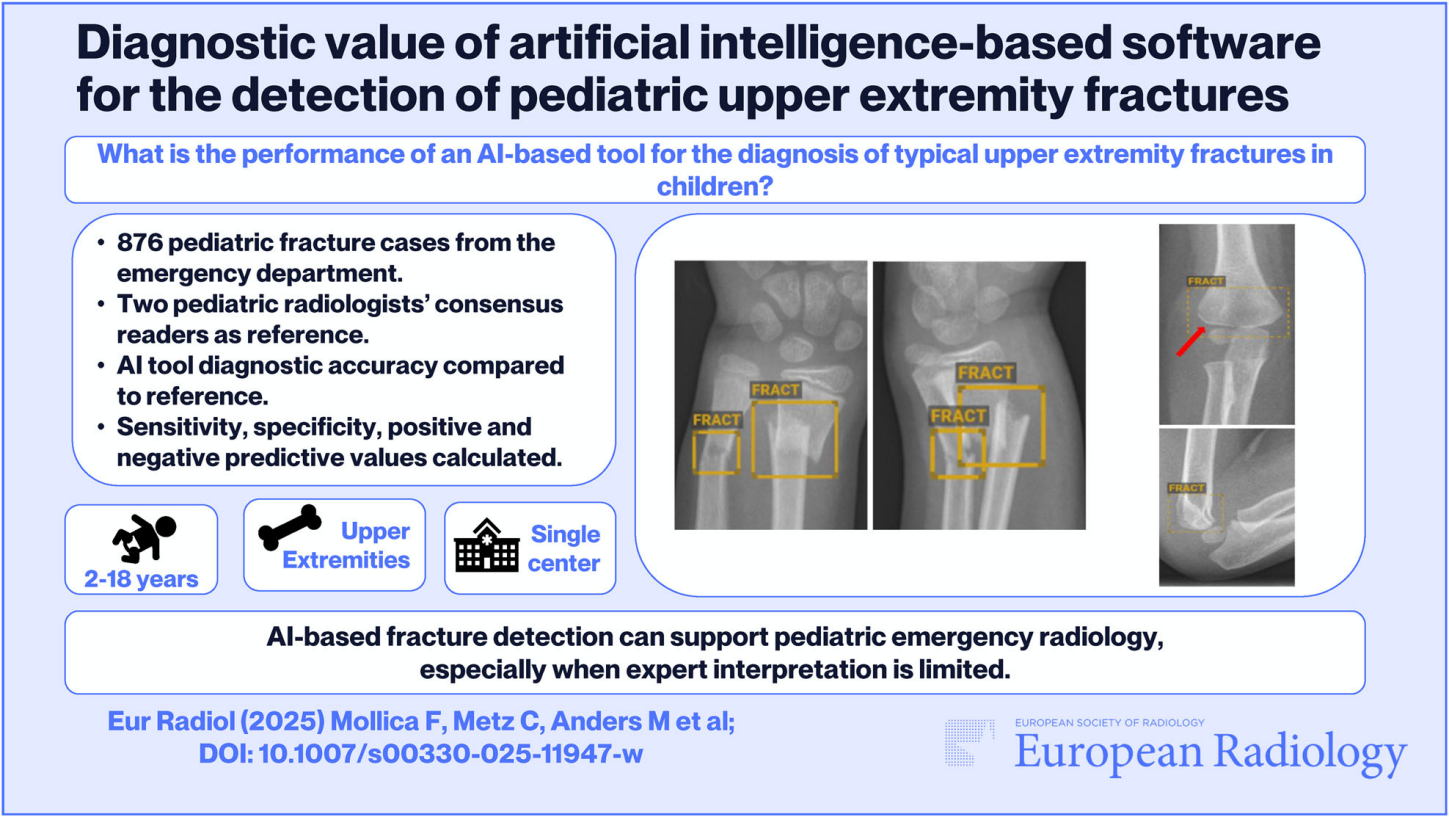

## Introduction

Fractures in children represent a common medical issue and are a leading cause of emergency room visits. Pediatric fractures differ substantially from those in adults due to their unique morphology, incidence, and mechanisms of injury, which are closely related to developmental stages and bone composition [1]. Among all fractures in children, more than 75% occur in the upper extremities, with the forearm being most frequently affected. Prompt and accurate fracture detection is critical in pediatric patients to avoid long-term complications, particularly given the dynamics of the growing skeleton [2, 3].

In standard clinical practice, suspected fractures are evaluated using conventional radiographs. Children are inherently more radiosensitive due to their higher proportion of dividing cells, their longer life expectancy, and their vulnerable immune and hormonal systems [4]. These factors necessitate the application of the “as low as reasonably achievable” (ALARA) principle to minimize radiation exposure [4]. The complexity of pediatric skeletal anatomy, such as the presence of growth plates and primary as well as secondary ossification centers, presents challenges for accurate interpretation of radiographs by general radiologists or emergency physicians, who may not be specialized in pediatric care or radiological imaging analysis. In this context, a lack of specialization may lead to CT diagnostics being used prematurely—thus disregarding the ALARA principle and possibly causing unnecessarily high radiation exposure. In this context, AI tools could be valuable as a supportive tool or second observer, especially when expertise in pediatric radiology is not readily available, and thus could aid in preventing CT or repeated radiographs in doubtful constellations [5]. Moreover, increasing workload in radiology departments and the shortage of pediatric radiology specialists exacerbate the challenges mentioned in pediatric imaging. A relevant number of missed fractures in emergency settings are attributed to such factors, highlighting the need for adjunctive diagnostic tools [6].

Over the past decade, artificial intelligence (AI) has emerged with the potential to enhance diagnostic accuracy, efficiency, and consistency in radiological image analysis [7, 8]. AI algorithms have shown great promise in automating image analysis, reducing processing time, recognizing patterns, and reducing observer-dependent result variability [9, 10]. BoneView® (Gleamer) is an AI-based tool specifically designed for the detection of bone fractures in conventional radiographs. While the software has demonstrated robust performance in adult populations [11, 12], its application in pediatrics requires further validation due to the unique characteristics of the pediatric skeleton described above. The aim of this study was to evaluate the diagnostic accuracy of this AI tool in detecting upper limb fractures in children aged 2 to 18 years.

## Materials and methods

### Study population and design

This is a retrospective observational study to evaluate the diagnostic accuracy of an AI-based software for the detection of pediatric fractures of the upper extremity. The AI software included a dedicated feature for detecting elbow joint effusions (EJE). This feature was limited to the elbow and not available for other joints. The presence of an EJE, as an indirect fracture sign, was included in the evaluation of the elbow images. The radiographic data analyzed in this study were collected from the database of a university pediatric radiology department. Ethical approval was granted by the local ethics committee, and the study adhered to the Declaration of Helsinki [13].

The study included children aged 2 to 18 years admitted to the pediatric emergency department with suspected traumatic fractures of the upper extremities. The retrospective data collection started from November 2023, consecutively backward (until July 2019), to collect at least 50 patients with a fracture in each defined anatomical region, regardless of patient age and sex (allowing additional analysis of patient characteristics for the fracture regions analyzed). Patients who suffered a fracture in more than one of the anatomical regions examined could be included more than once, leading to a total number of more than 50 patients for those localizations where fractures are frequently associated with concomitant fractures. Inclusion criteria were an initial radiographic evaluation for acute trauma and patient age between 2 and 18 years. Exclusion criteria were follow-up imaging for previously diagnosed fractures, pathological fractures, non-accidental injury patterns or stigmatic fractures such as bucket-handle fractures that are commonly outcomes of child abuse [14], and a lack of agreement between the initial report and the re-evaluation of the radiographs by a study radiologist (see below). Patients with incomplete imaging were also excluded from the analysis. Imaging included single-plane radiographs for the clavicle and bi-plane radiographs for other upper extremity regions. Radiographs were included despite minor technical limitations such as small malrotations due to pain or movement restrictions due to injuries, if they were still evaluable regarding the clinical question. Based on the initial pediatric radiology report, which was re-evaluated by a study radiologist as part of this research project, images were assigned to the categories “positive” and “negative” for the presence of a fracture or an EJE, respectively.

### AI software

BoneView® (Gleamer) software was originally developed using a set of 60,170 trauma radiographs acquired from 22 institutions [15]. Its current version employs a deep learning algorithm trained on over 300,000 radiographs (30% pediatric) provided by more than 60 medical institutions [16]. The software uses a two-stage object detection framework that combines Feature Pyramid Networks and Region Proposal Networks to identify fractures, their dislocations, and EJE [17]. Regarding fractures, the AI tool outputs include trinary classifications: “yes” (fracture is present) or “no” (fracture is not present) or “doubt” (fracture is possible), the latter for uncertain findings requiring additional review. For EJE and fracture dislocations, the output is binary: “yes” or “no.” The technical specifications of the algorithm have been published elsewhere [16].

### Analysis of the diagnostic accuracy of the AI software

All radiographs were evaluated by pediatric radiologists of the Pediatric Radiology Department with at least 5 years of pediatric radiology experience, and the corresponding reports were included from the Radiology Information System. Each included dataset was re-evaluated, blinded to the initial radiological report, by a second radiologist with 5 years of experience in pediatric radiology. Only matching diagnoses between the initial report and the re-evaluation were accepted as a reference standard for assessing the performance of the AI software. If the resulting diagnoses did not match, images were excluded from further analyses. Included radiographs were grouped according to the body region, i.e., clavicle, shoulder, upper arm, elbow, forearm, wrist, hand, and fingers.

**Fig. 1:**
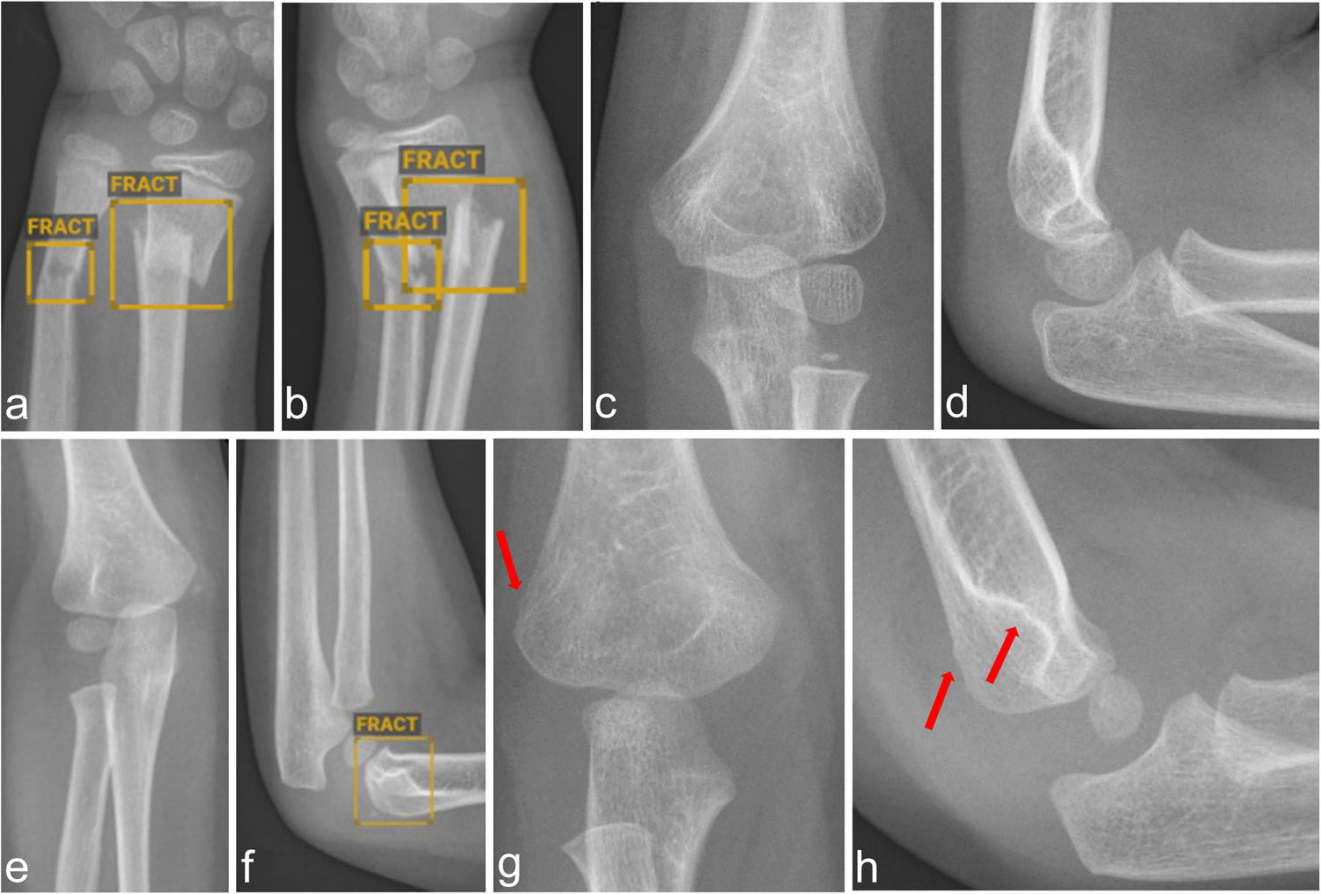
**a**, **b** Example of a correct-positive marking of a forearm fracture by the AI. **c**, **d** Example of the correct negative evaluation of a left elbow by the AI. **e**, **f** Example of a false-positive marking of a right supracondylar fracture by the AI. **g**, **h** Example of a false-negative evaluation of an elbow with supracondylar humeral fracture (arrows), soft tissue swelling and positive fat pad sign

**Fig. 2:**
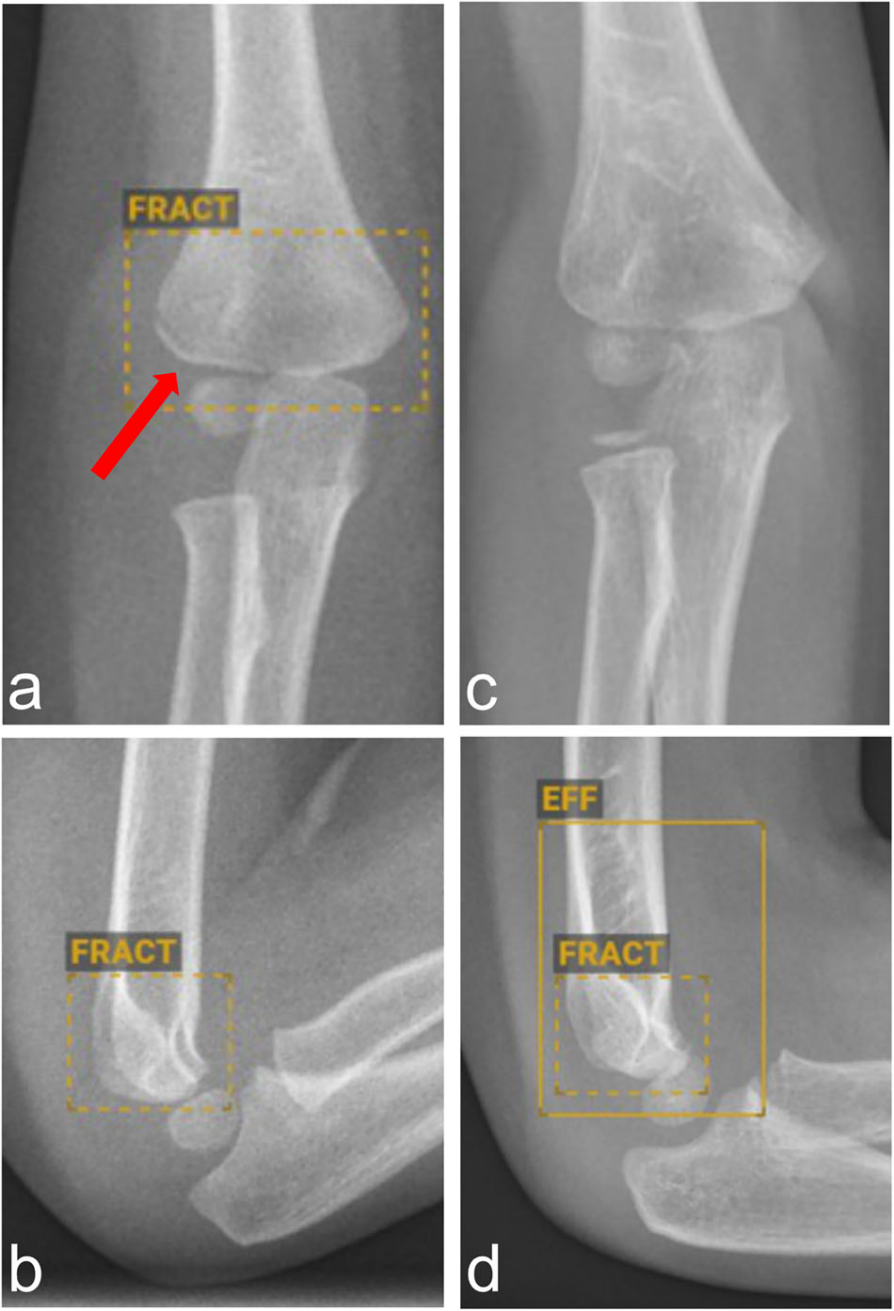
**a**, **b** Example of a correct “doubt” marking of an elbow joint with a fracture of the lateral humeral condyle (arrow) by the AI. In contrast to positive fracture markings, fracture markings indicating the “doubt” category are dashed. **c**, **d** Example of an incorrect “doubt” marking of a right elbow, where no fracture is present

It was then checked whether the AI software had recognized a fracture or not. Depending on agreement with the reference standard (assessment by the pediatric radiologists), radiographs were categorized into the groups true-positive, false-positive, true-negative, and false-negative. If a child had several fractures in one of the defined body regions, these were combined and assessed as one case. Only if the AI software program correctly identified all fractures was the case classified as true-positive. If the AI software program identified a fracture in an incorrect location in addition to a correct marking, the case was classified as false-positive. Figure 1 shows exemplary cases of all categories. If a radiograph was marked as “doubt” by the AI, it was assessed as having a fracture (Fig. 2). An analogous procedure was chosen for the analysis of the AI software regarding the detection of EJE and the findings of the software were compared with the reference standard, including subsequent classification into correct-positive cases, etc.

### Statistical analysis

Statistical analyses were conducted using IBM SPSS Statistics (Version 29). Regarding analysis of the diagnostic accuracy of the AI-based software with the evaluation of the pediatric radiologists as reference, sensitivity, specificity, positive predictive value (PPV), and negative predictive values (NPV) were calculated across the 8 anatomical regions.

## Results

### Patient characteristics

A total of 826 patients met the inclusion criteria, resulting in 876 cases, as 42 patients included suffered fractures in 2 different body regions and 4 patients in 3 body regions, respectively. Table 1 summarizes the demographic and clinical characteristics of the study population. Fractures were confirmed in 452 cases (52%), while 424 cases (48%) had no fractures. The characteristics of the study subpopulations for each fracture site are shown in Table 1. Among the consecutively included 452 patients with upper extremity fractures, a higher incidence was observed in children under 8 years of age (7.6 ± 4.1 years), with boys accounting for the majority of cases (*n* = 293; 65%).

**Table 1:** Case characteristics from the included 826 patients

Region	No. of cases	Male (%)–Female (%)	Average age, years (SD)	Fracture (%)–No fracture
Total	876	555 (63%)–321 (37%)	9.2 (± 4.4)	452 (51%)–424
Clavicle	104	70 (67%)–34 (33%)	7.7 (± 5)	54 (52%)–50
Shoulder	110	71 (64%)–39 (36%)	10.9 (± 3.7)	52 (47%)–58
Upper arm	102	63 (62%)–39 (38%)	8.7 (± 4.1)	50 (49%)–52
Elbow	102	48 (47%)–54 (53%)	6.8 (± 3.9)	54 (53%)–48
Forearm	105	60 (57%)–45 (43%)	7.4 (± 4)	55 (52%)–50
Wrist	126	87 (69%)–39 (31%)	10.5 (± 3.5)	77 (61%)–49
Hand	106	82 (77%)–24 (23%)	11.9 (± 4)	50 (47%)–56
Fingers	121	74 (61%)–47 (38%)	9.2 (± 4.2)	60 (49%)–61

### Diagnostic accuracy of the AI-based fracture detection

Based on the entire set of radiographs analyzed and on pediatric radiologists as reference, overall sensitivity was 89% and specificity was 91%.

Subgroup analyses for the different anatomical regions revealed substantial differences in diagnostic accuracy, which are reported in detail in Table 2. The best diagnostic accuracy was observed at the wrist with a sensitivity of 96% and specificity of 94%. The lowest sensitivity was observed for fractures of the fingers (73%), while the lowest specificity was found for the elbow fractures (65%, Table 2).

**Table 2:** Statistics of fracture analyses, all values are in percent

Anatomical region	Sensitivity	Specificity	PPV	NPV
Total	89	91		
Clavicle	92	94	94	92
Shoulder	88	89	88	90
Upper arm	92	85	85	92
Elbow	87	65	73	81
Forearm	96	92	93	96
Wrist	96	94	96	94
Hand	86	87	86	87
Fingers	73	92	90	78

*NPV* negative predictive value, *PPV* positive predictive value

### Elbow joint effusion

Using the assessment by the pediatric radiologists as a reference, the AI-based software performed worse. The sensitivity in detecting an EJE was 79%, specificity was 51%.

## Discussion

The AI system demonstrated good overall performance in evaluating radiographs of the pediatric upper extremity regarding the presence of fractures, with an overall accuracy rate of 95%, sensitivity of 89%, and specificity of 91%. However, the overall performance was significantly lower than the evaluation by pediatric radiologists. Variations in performance were observed depending on fracture location. No significant differences from the pediatric radiologist evaluation were found for radiographs of the clavicle, upper arm, forearm, and wrist. In contrast, significant lower performance was identified in the assessment of the shoulder, elbow, hand, and fingers. The AI tool achieved its best performance in the evaluation of the wrist, followed closely by the clavicle. The worst performance was observed in the evaluation of the elbow. The AI software demonstrated particularly poor performance in identifying EJE, with a mean accuracy of 62%, sensitivity of 79%, and specificity of 51%.

The results of this study highlight the diagnostic potential of AI tools in the context of pediatric radiology and their ability to reliably identify fractures while minimizing false positives. However, the performance of this software tool was highly dependent on the location of the fractures. These results align with previous studies indicating the efficacy of AI in musculoskeletal radiology [11, 12, 18].

A key strength of this AI software lies in its performance for clavicle, forearm, and wrist fractures, where it achieved sensitivity and specificity values exceeding 90%. This is consistent with existing literature, which emphasizes the potential of AI to enhance diagnostic workflows in areas with high fracture prevalence [16]. In contrast, the software’s sensitivity for finger and elbow fractures was lower, at 73% and 87%, respectively. This discrepancy underlines the challenges associated with anatomical variations and overlapping structures in pediatric radiographs, as noted in earlier studies [5, 14]. AI tools could serve as valuable adjuncts to clinical decision-making by reducing false-positive diagnoses, thereby minimizing unnecessary follow-up imaging and associated costs. Previous studies have similarly reported the potential of AI to improve diagnostic accuracy while reducing observer variability [17, 19].

Despite its overall robust performance, BoneView® demonstrated limitations in detecting EJE. False-positive EJE detections were frequently associated with hypertranslucent soft tissue areas, particularly around the elbow. These findings align with prior studies showing how difficult it is for AI tools to assess soft tissue abnormalities in radiographs [20]. Addressing those limitations, being specific for the pediatric population, will be essential for optimizing the software’s utility in clinical practice in pediatric radiology.

The results reveal important implications for healthcare systems facing resource constraints. In regions with limited access to pediatric radiology specialists, AI tools could bridge gaps in care by providing rapid, reliable fracture detection, even more so if they are specifically developed for use in children and adolescents in the future.

While our study primarily contributes to the assessment of the diagnostic accuracy of a specific AI tool for pediatric upper extremity fracture detection, it is crucial to acknowledge that this does not fully capture clinical utility. Real-world benefits of AI implementation require further evidence demonstrated by improved reader performance, enhanced patient outcomes, and cost-effectiveness in clinical practice. Future research should focus on prospective studies evaluating how the integration of AI tools for fracture analysis impacts pediatric radiologists’ workflow efficiency, as well as longitudinal studies assessing patient health outcomes and economic analyses to establish cost savings. We already know from existing literature that AI-assisted diagnostics can reduce healthcare costs and improve outcomes across different medical fields by enabling earlier and more accurate diagnoses, but these benefits depend on validation in specific clinical contexts and populations such as pediatric radiology [21–23].

Our study contributes to previous research evaluating AI-based fracture detection in children by focusing specifically on upper extremity fractures within a large pediatric population. While several studies have demonstrated strong diagnostic performance of BoneView and other AI models for appendicular fractures in pediatric cohorts [5, 24] our work is distinguished by its comprehensive anatomical sub analysis of the upper extremities and its inclusion of EJE detection as part of the evaluation. Unlike prior studies that included smaller or more heterogeneous samples, our large, single-center dataset allows for assessment of the AI software’s performance across various upper limb regions [25]. Additionally, by analyzing the clinical utility of EJE detection, a feature not assessed in previous research, we provide new insight into the potential role of AI in supporting pediatric trauma/fracture care.

The retrospective design of this study may lead to selection bias, as the dataset was limited to cases presenting at a single tertiary care center. We have not systematically documented the number of patients screened and excluded at each stage of the selection process and are therefore unable to provide an exact number of exclusions based on specific criteria. The performance of the AI software was assessed in comparison to the evaluations of pediatric radiologists, with the assumption that their interpretations were 100% accurate. While this assumption could have potentially limited and underestimated the AI software’s performance, the fact that the AI’s sensitivity and specificity never reached 100% suggests that its evaluation was not constrained by this comparison. To establish a robust reference standard (“truth”), we excluded cases in which the two pediatric radiologists disagreed. By including only cases with concordant interpretations, we aimed to reduce reference uncertainty and provide a more consistent basis for evaluating AI’s performance. Nonetheless, we recognize that omitting cases that might have been subsequently confirmed by follow-up imaging could introduce bias, particularly if these excluded cases involved diagnostically challenging or rare fractures. This limitation may impact the external validity and generalizability of our findings. To focus the software analysis on traumatic fractures only, we tried to exclude fractures of non-traumatic etiology, e.g., abuse-associated fractures. However, we do recognize the possibility that some cases of fractures resulting from child abuse may have been included in our dataset due to nonspecific fracture types. Finally, while BoneView® performed well in most regions, further validation studies using larger, multi-center datasets are necessary. We also acknowledge that our findings are limited to the application of this specific AI software only and that different outcomes may be obtained using other AI tools available. While the exclusive focus on upper extremity fractures represents the novelty of this study, it also introduces a limitation. The findings and conclusions are specific to upper extremity fractures in the pediatric population. This should be considered when attempting to generalize results to fractures in other anatomical regions in the pediatric population.

## Conclusions

This study demonstrates the potential of BoneView® as an assistive diagnostic tool in pediatric radiology. Our findings align with the reported performance of the AI software in an adult population [15]. The software provides overall high sensitivity and specificity, particularly for pediatric fractures of the clavicle, the forearm, and the wrist. However, there are still challenges at certain anatomical sites, in the detection of complex or very subtle fractures and soft tissue abnormalities, such as EJE. To fully exploit the advantages of AI in pediatric radiology, the underlying algorithms must be further developed, considering the special characteristics of the growing skeleton. However, software solutions like BoneView® can be helpful, especially in resource-constrained settings, where AI tools can bridge gaps in the availability of specialists. By integrating AI tools into routine clinical practice, healthcare providers could enhance diagnostic accuracy, streamline workflows, and address critical gaps in specialist radiology expertise.

## Abbreviations

AI: Artificial intelligence
ALARA: As low as reasonably achievable
EJE: Elbow joint effusion
NPV: Negative predictive value
PPV: Positive predictive value
